# Near-100 MeV protons via a laser-driven transparency-enhanced hybrid acceleration scheme

**DOI:** 10.1038/s41467-018-03063-9

**Published:** 2018-02-20

**Authors:** A. Higginson, R. J. Gray, M. King, R. J. Dance, S. D. R. Williamson, N. M. H. Butler, R. Wilson, R. Capdessus, C. Armstrong, J. S. Green, S. J. Hawkes, P. Martin, W. Q. Wei, S. R. Mirfayzi, X. H. Yuan, S. Kar, M. Borghesi, R. J. Clarke, D. Neely, P. McKenna

**Affiliations:** 1 SUPA Department of Physics, University of Strathclyde, Glasgow, G4 0NG UK; 2 Central Laser Facility, STFC Rutherford Appleton Laboratory, Oxfordshire, OX11 0QX UK; 3 Centre for Plasma Physics, School of Mathematics and Physics, Queen’s University Belfast, Belfast, BT7 1NN UK; 4 Key Laboratory for Laser Plasmas and CICIFSA, School of Physics and Astronomy, Shanghai Jiao Tong University, Shanghai, 200240 China

## Abstract

The range of potential applications of compact laser-plasma ion sources motivates the development of new acceleration schemes to increase achievable ion energies and conversion efficiencies. Whilst the evolving nature of laser-plasma interactions can limit the effectiveness of individual acceleration mechanisms, it can also enable the development of hybrid schemes, allowing additional degrees of control on the properties of the resulting ion beam. Here we report on an experimental demonstration of efficient proton acceleration to energies exceeding 94 MeV via a hybrid scheme of radiation pressure-sheath acceleration in an ultrathin foil irradiated by a linearly polarised laser pulse. This occurs via a double-peaked electrostatic field structure, which, at an optimum foil thickness, is significantly enhanced by relativistic transparency and an associated jet of super-thermal electrons. The range of parameters over which this hybrid scenario occurs is discussed and implications for ion acceleration driven by next-generation, multi-petawatt laser facilities are explored.

## Abstract (web summary)

It is a challenge to scale up laser-ion acceleration to higher ion energies. Here the authors demonstrate a hybrid acceleration scheme based on the relativistic induced transparency mechanism using linearly polarised laser interaction with foil targets and its future implication in using high power lasers.

## Introduction

The potential to produce compact sources of energetic (tens to hundreds of MeV per nucleon) ions with unique beam properties, including short temporal duration, motivates an intense international research activity in high-power laser-driven ion acceleration^1,2^. These enabling sources are being applied for radiographic density diagnosis with micron-scale resolution^3^, for probing highly transient electric and magnetic fields in plasmas with picosecond resolution^4^, for the isochoric heating of matter^5^ and for probing radiation-induced processes in matter^6^. Societal applications, such as in biomedicine (for example, hadron therapy^7^) and fusion energy (for example, fast ignition of fusion targets^8^), have also been proposed. Some of the applications require higher ion energies than presently achieved, and many require high laser-to-ion energy conversion efficiency, as well as spectral and beam divergence control.

Efforts to increase the maximum ion energy have largely focused on the development of novel acceleration mechanisms involving ultrathin (tens to hundreds of nanometres) foil targets. To date, the most widely investigated ion acceleration mechanism is the target normal sheath acceleration (TNSA)^9^ scheme. This proceeds via the build-up of TV m^−1^ electrostatic fields at the rear side of a thin target foil, produced by relativistic electrons generated at the laser-irradiated side, typically via the oscillating **j** × **B** heating of linearly polarised light. TNSA proton energies in excess of 85 MeV from ultrathin foils have recently been reported^10^. A different approach, based on using the radiation pressure of the laser light has also been explored. At sufficiently high radiation pressure, the laser pulse pushes the plasma electrons inwards, establishing a strong electrostatic field and resulting in the radiation pressure acceleration (RPA) of ions. Hole-Boring^11^ and Light-Sail^12^ are two specific modes of this acceleration mechanism, which has been shown to result in peaked ion energy spectra^13^. RPA benefits from the use of circularly polarised light due to the absence of the oscillating **j** × **B** heating^14^ and this has recently been demonstrated experimentally^15^. The polarisation dependence of RPA has also been used to attribute this mechanism to measurements of high-energy protons with a broad energy spectrum^16^. By contrast, the use of linearly polarised light is predicted to result in the formation of a dual-peaked electrostatic field, due to RPA at the target front and TNSA at the rear^17^, and hybrid scenarios in which ions from both mechanisms combine^18^.

Whereas ion acceleration is particularly effective in ultrathin foils, such targets are subject to the onset of relativistic induced transparency (RIT)^19^. RIT occurs if $a_{\mathrm{0}} \gg \omega _{\mathrm{p}}^2\ell {\mathrm{/}}2c\omega _{\mathrm{L}}$, where *ω*_L_ and *ω*_p_ are the laser and plasma frequency, respectively, *c* is the speed of light in vacuum, $\ell$ is the target thickness and *a*_0_ is the normalised light amplitude^19^. At presently achievable intensities, RIT typically occurs due to the combined effects of plasma expansion and a relativistic increase in the electron mass, which act to decrease *ω*_p_. Propagation of the remainder of the laser pulse through the target results in several phenomena that can strongly affect ion acceleration. In the case of short, high contrast pulses, RIT produces a relativistic plasma aperture in the foil, resulting in diffraction of the transmitted laser light^20^. This modulates the electrostatic acceleration field and thus the spatial distribution of the resulting proton beam^21^. Volumetric heating by the transmitted laser light has been shown to enhance the energy of TNSA ions^22^. Energy exchange via streaming plasma instabilities has also been numerically explored under these conditions^23,24^ and is invoked in an acceleration scheme called break-out afterburner^25^. In the same ultrathin-foil-RIT regime, driven by relatively long (hundreds of femtoseconds) and linearly polarised laser pulses, Powell et al.^26^ reports that a relativistic plasma jet is produced, which locally enhances the maximum proton energy. Similar jet structures are reported in Palaniyappan et al.^27^ to explain measured energy enhancements and narrow energy spread features in Al and C ions.

Given the importance of ultrathin foils as ion acceleration targets, it is imperative that we improve our understanding of the coupling between acceleration mechanisms and the collective particle and field dynamics driven by transparency in this complex interaction regime. This is also particularly pertinent to future investigations of ion acceleration using next-generation, multi-petawatt laser facilities presently under development, such as the Extreme Light Infrastructure^28,29^, Apollon^30^ and XCELS^31^. Difficulties in converting the inherent linearly polarised light to circular polarisation (due to the large diameter of the unfocused beam) means that linearly polarised-driven hybrid acceleration scenarios are highly likely to prevail. In addition, these facilities are expected to produce focussed intensities up to 10^23^ W cm^−2^, which would render near-solid density foils relativistically transparent, increasing the role of RIT-enhanced processes.

Here we report on an experimental and numerical investigation of proton acceleration from ultrathin foils irradiated by relatively long pulses of linearly polarised laser light. Efficient acceleration to energies exceeding 94 MeV is demonstrated. Particle-in-cell (PIC) simulations reveal that this is driven by a dual-peaked electrostatic field, producing a hybrid RPA-TNSA scenario, and that for optimum target thicknesses it is enhanced by the onset of RIT. The proton energies are further boosted, in a narrow angular range, by a RIT-driven jet of super-thermal electrons. Whereas this scheme prevails for long pulses, modelling suggests that RPA-dominant acceleration regimes, with and without RIT enhancement, are achievable with short pulses. Simulations for pulse parameters within the range expected at next-generation, multi-petawatt laser facilities indicate that through choice of target thickness the enhanced hybrid acceleration scheme can be optimised for either high energy or for the production of a narrow-band spectral peak.

## Results

### Optimisation of proton acceleration with foil thickness

**Fig. 1:**
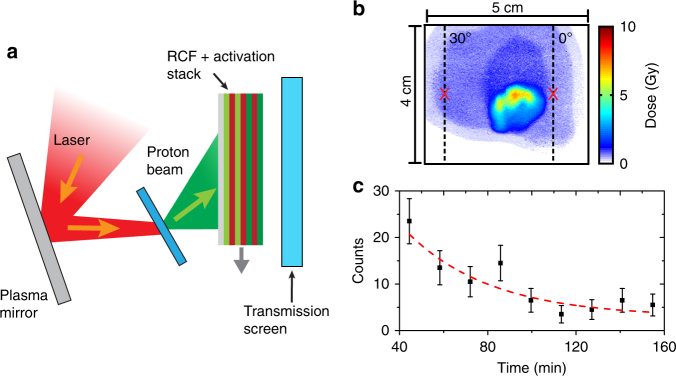
Schematic of the experiment set-up and example measurements. **a** A pulse from the Vulcan laser is focused by an *f/*3 off-axis parabola and reflected from a planar plasma mirror onto a target foil. The spatial-intensity profile of the beam of accelerated protons is measured using stacked dosimetry film (RCF), interwoven with Cu foils for nuclear activation measurements. The transmitted laser energy is characterised on a transmission screen (with the stack retracted). **b** Example proton beam dose distribution, as measured using RCF, for proton energies *ε*_p_ ≥ 89 MeV. The red markers at 0° and 30° correspond to the laser axis and target normal axis, respectively. **c** Example measurements of the positron-emission decay of the ^63^Zn radioisotope produced by proton activation of Cu in the stack (^63^Cu(p,n)^63^Zn), for protons with *ε*_p_ > 92 MeV. The time is measured from the time of the laser-plasma interaction and the error bars are determined from the statistical uncertainties in the measured counts. The dashed curve is a fit corresponding to the 38.5 min half-life of ^63^Zn, confirming proton-induced activation in the high-energy region of the filter stack

Figure 1a, b shows the experiment set-up (discussed in the Methods section) and an example beam profile for proton energy (*ε*_p_) ≥ 89 MeV, respectively. The high proton energies detected using stacked dosimetry film (radiochromic film; RCF) were confirmed by measurement of proton-induced activation of Cu filters interspersed with the film. Specifically, the ^63^Cu(p,n)^63^Zn reaction channel was verified by measuring the 38.5 min half-life of the positron-emitting ^63^Zn daughter radioisotope, using a NaI detector coincidence system to detect the 511 keV photons created upon positron annihilation^32^. An example measurement corresponding to *ε*_p_ > 92 MeV is shown in Fig. 1c. The highest proton energy was between 94 and 101 MeV—strong signal was measured on the 94 MeV RCF layer, but not on the next, 101 MeV, layer. This was achieved for a plastic target foil, with thickness, $\ell$, equal to 90 nm.

**Fig. 2:**
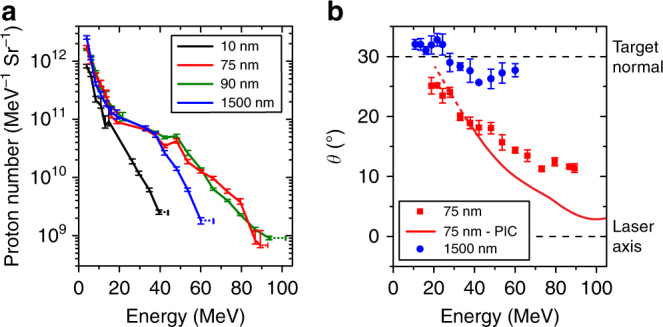
Measurements of proton beam spectrum and direction. **a** Example proton energy spectra, as deconvolved from the RCF measurements, for given foil thickness, $\ell$. The horizontal error bars at the maximum energy are defined by the energy corresponding to the last RCF layer for which proton signal is measured (lower limit) and the energy of the next RCF layer (upper limit). The vertical error bars are defined by the level of uncertainty in the calibration of the RCF. **b** Measured angle of the centre of the proton beam, *θ*, with respect to the laser axis (in the plane of the incident laser beam), as a function of energy, for $\ell$ = 75 nm (red) and 1.5 μm (blue). The error bars are defined by the uncertainty in determining the angle of the centre of the proton beam from application of a beam fitting routine. An example PIC simulation result for $\ell$ = 75 nm (red curve) is included for comparison. The dashed lines mark the target normal and laser axis, for ease of reference

Representative spectra (sampled over the whole proton beam) for four target thicknesses and an example of the proton beam direction (beam centre) as a function of energy are shown in Fig. 2a, b, respectively. The high-energy component steers towards the laser axis in the case of targets undergoing RIT, as discussed below. The fraction of light transmitted through the target was measured using a calibrated uniform scatter (Spectralon) screen, for separate shots with the RCF stack removed. The velocity of the critical density (*n*_crit_) surface (at which *ω*_p_ = *ω*_L_) was determined by measuring the Doppler shift of the backscattered second harmonic light produced near the peak of the laser intensity.

**Fig. 3:**
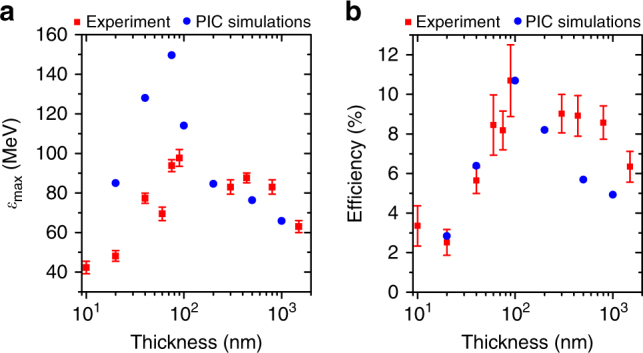
Scaling of the maximum proton energy and energy conversion efficiency with thickness. Measured **a** maximum proton energy (*ε*_max_) and **b** laser-to-proton energy conversion efficiency (*η*), as a function of foil thickness (red), together with results from 2D PIC simulations (blue). The conversion efficiencies from the simulations are scaled by a fixed value, such that the maximum value is normalised to the measured maximum efficiency. The maximum energies from the simulations are unscaled. Both proton beam parameters are maximised at an optimum target thickness range of 70–100 nm. The error bars in the maximum energy are defined by the energy corresponding to the last RCF layer for which proton signal is measured (lower limit) and the energy of the next RCF layer (upper limit). The error bars in the conversion efficiency are determined from the uncertainties in the measured proton energy spectra

**Fig. 4:**
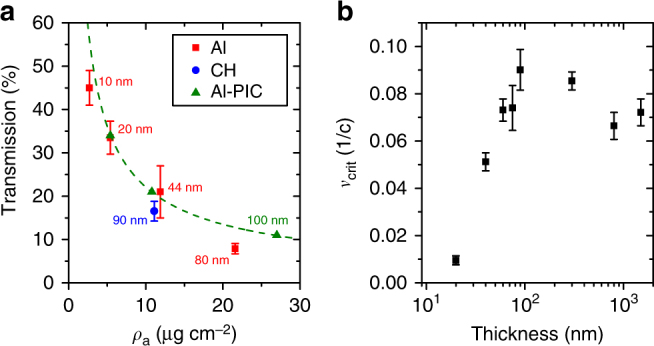
Measurements of laser light transmission and target critical surface velocity. **a** Percentage of laser light transmitted as a function of target areal density (*ρ*_a_) for stated thicknesses ($\ell$), for plastic (blue; CH) and Al (red) foils. The error bars are determined from the uncertainties in the calibration of the light level on the transmission screen and the area of the transmitted beam sampled. Results from the 2D PIC simulations with Al foils (green; Al-PIC) are included. **b** Recession velocity of the critical density surface (*v*_crit_) as a function of $\ell$ for plastic targets, as determined from measurements of spectral shift in second harmonic light produced at the critical density surface. The lower and upper limits of the error bars are determined from the maximum red-shifted wavelength, where the signal is resolvable above the noise level and by application of a fitting routine, respectively

The measured maximum proton energy, *ε*_max_, and laser-to-proton energy conversion efficiency, *η*, are plotted as a function of $\ell$ in Fig. 3a, b, respectively, alongside results from two-dimensional (2D) PIC simulations (discussed below). In the range $\ell$ > 170 nm, the results agree with measurements reported in Wagner et al.^10^, with similar plastic targets and 0.5–0.8 ps duration, (0.7–2.6) × 10^20^ W cm^−2^ laser pulses. *ε*_max_ and *η* are maximised at an optimum thickness range, $\ell _{{\mathrm{opt}}}$ ~ 70–100 nm. It is in this range that the target undergoes RIT near the peak of the laser intensity. Figure 4a shows the measured degree of laser light transmission as a function of target areal density, *ρ*_a_ (to facilitate comparison of Al and plastic foils), which increases with decreasing $\ell$. For $\ell$ < 100 nm, the magnitude of both *ε*_max_ and *η* decrease with increasing transmission. Furthermore, as shown in Fig. 4b, the recession velocity of the plasma critical density, driven by radiation pressure, decreases with increasing transmission, as expected. Thus, whereas the results suggest that the measured enhancement in proton acceleration is concurrent with some degree of transparency occurring, it is clear that significant transmission of the laser energy (due to the onset of RIT too early in the interaction) is detrimental to proton acceleration.

### Simulations of hybrid acceleration and transparency enhancement

**Fig. 5:**
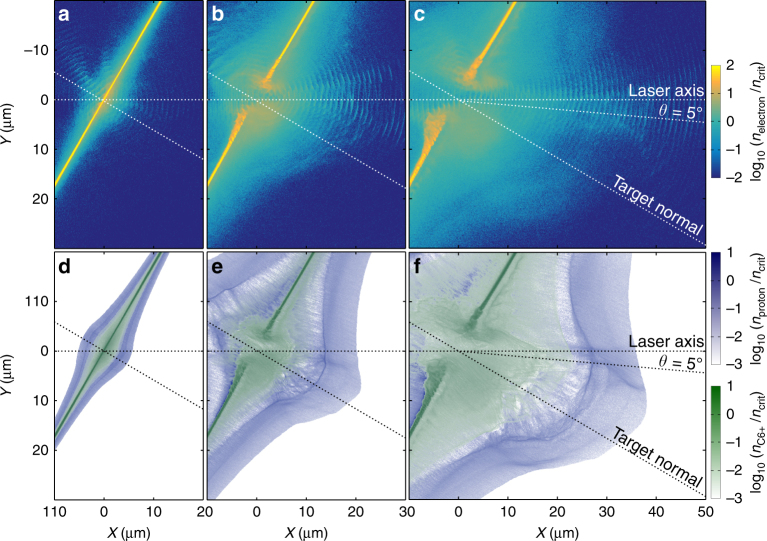
Simulation results displaying the electron and ion density spatial profiles. **a**–**c** Electron density profile at **a** *t* = −0.2 ps, **b** *t* = 0.1 ps and **c** *t* = 0.3 ps (where time *t* = 0 corresponds to the peak of the pulse arriving at the target). **d**–**f** Corresponding plots showing the proton (blue) and C^6+^ (green) ion density profiles

To investigate the underlying physics, we performed 2D PIC simulations using the fully relativistic EPOCH code^33^. As shown in Fig. 3, the results are very similar in terms of overall trend to those of the experiment, including the optimum $\ell$ for maximising *ε*_p_ and *η* (the absolute values are higher than those measured due to the 2D dimensionality of the simulations). To provide an overview of the overall behaviour, Fig. 5 shows example electron, proton and C^6+^ ion density distributions at three snapshots in time, for an optimum target thickness case, $\ell$ = 75 nm. Early in the simulation the target expands due to the TNSA mechanism. Ions with the highest charge-to-mass ratio gain most energy in the sheath field, resulting in the ion species becoming layered, with the protons at the front^26^, and a buffering effect between the carbon and proton ions, similar to that reported in ref. ^34^. As there is minimal overlap between the protons and carbon ions, there is negligible electron-screened Coulomb repulsion effects, such as reported in Liu et al.^35^ for the case of circularly polarised laser pulses. As the laser intensity increases, the radiation pressure induces an electric field component that accelerates a population of both the protons and carbon ions forward. At time *t* = −0.15 ps (where *t* = 0 ps corresponds to the peak of the pulse interacting with the target), the electron density along the laser propagation axis decreases below the relativistically corrected critical density and the target becomes relativistically transparent. As the laser propagates through the remainder of the target volume, it can directly accelerate electrons, forming a high-energy electron jet, as reported in ref. ^26^. The jet extends through and beyond the TNSA ion front. The highest energy proton bunch is observed in the vicinity of the electron jet. There may also be some additional energy transfer to the carbon ions, but they do not achieve sufficient energy to influence the highest energy protons.

**Fig. 6:**
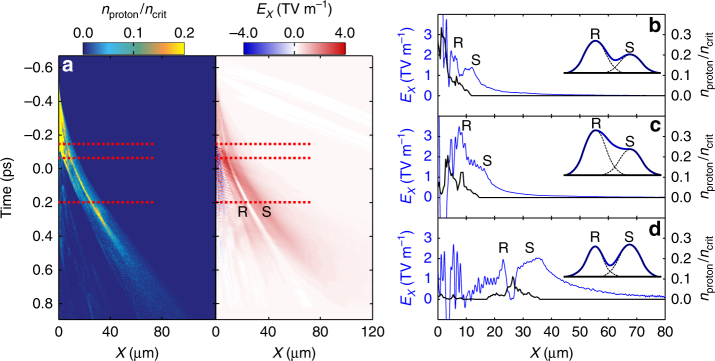
Simulation results illustrating the hybrid acceleration scheme. **a** Proton density (*n*_proton_) and longitudinal electrostatic field (*E*_*X*_; along the laser axis) as a function of *X* and time (where time *t* = 0 corresponds to the peak of the pulse arriving at the target) for the interaction of a 0.4 ps, 2 × 10^20^ W cm^−2^ laser pulse with a $\ell$ = 75 nm plastic foil. The three dotted lines correspond to the example times considered in **b**–**d**, from top to bottom, respectively. **b**–**d** Longitudinal electric field (blue) and proton density (black) at **b** *t* = −0.15 ps, **c** *t* = −0.05 ps and **d** *t* = 0.2 ps. Peaks R and S are the RPA and TNSA fields, respectively. The insets in **b**–**d** schematically illustrate the evolution of the dual-peaked structure

**Fig. 7:**
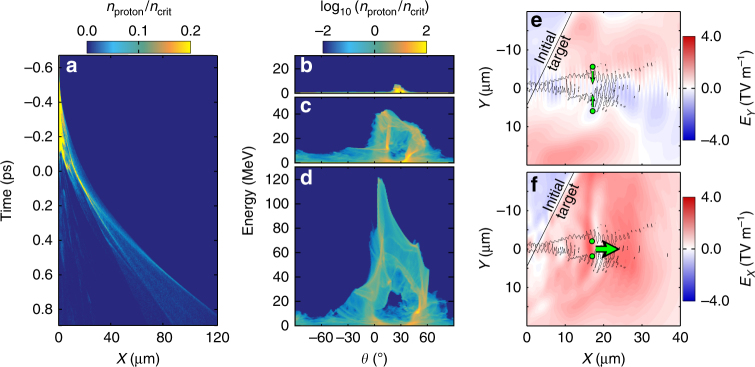
Simulation results illustrating the influence of the electron jet on the proton beam. **a** Evolution of the proton density (*n*_proton_) at angle *θ* = 5° (same simulation as in Fig. 6). **b**–**d** Proton density and energy as a function of angle *θ* (with respect to the laser axis) at **b** *t* = −0.3 ps, **c** *t* = 0.1 ps and **d** *t* = 0.5 ps. **e** Transverse electric field (*E*_*Y*_) with super-thermal electron jet, as shown by contours for electrons with >10 MeV at 0.1*n*_crit_. The arrows show the direction of the resultant force on protons, as represented by the green dots. **f**, Same for the longitudinal electrostatic field (*E*_*X*_)

Next, the evolution of the electrostatic fields and the proton density is investigated in order to understand the process by which *ε*_p_ is enhanced (again for an optimum case of $\ell$ = 75 nm). The results are shown in Figs. 6 and 7. First we consider the main acceleration mechanisms occurring. Figure 6a shows the proton density and corresponding longitudinal electrostatic field, along the laser axis, as a function of time. The acceleration is dominated by a moving dual-peaked electrostatic field, similar to that investigated numerically in ref. ^17^. The peak labelled S is the TNSA sheath field, generated at the target rear early in the interaction. Peak R is produced slightly later by the laser radiation pressure at the front (resulting in RPA). Line-outs at three example times, corresponding to the dashed lines in Fig. 6a, are presented in Fig. 6b–d. Protons accelerated by peak R catch up with those accelerated by S, producing a single bunch between the peaks, which is accelerated by the hybrid RPA-TNSA scheme, as introduced by Qiao et al. ^18^. However, unlike the process described in that paper, the peak eventually broadens out at late times when the dual-field structure decays.

The increased heating of the plasma electrons by the portion of the laser pulse, which propagates through the expanding foil, due to RIT, enhances the magnitude of both peaks in the longitudinal electrostatic field. This occurs in a transient way, in that peak R is enhanced first (by almost a factor of 2), which temporarily results in a merging of the field structures, as shown in Fig. 6c. As the laser pulse propagates forward, peak S then increases in magnitude and the space charge of the proton bunch suppresses the field between the peaks as the interaction evolves (Fig. 6d). The dual peak structure with bunched proton population continues until the overall field decays.

The highest-energy protons are observed at *θ* ~ 5°, where *θ* is the angle with respect to the laser propagation axis. Figure 7a shows the proton density-time plot at this angle. When compared to the example results in Fig. 6a, it is clear that part of the proton bunch is accelerated to higher energies. This is also clearly observed in the *ε*_p_-*θ* plots shown at three example times in Fig. 7b–d. Early in the interaction, the proton beam is centred on the target normal axis (*θ* = 30°). As the simulation evolves, a ring-like density distribution emerges at relatively low proton energies, similar to that discussed in refs. ^34,36^. After the onset of RIT occurs, part of the proton population (in the angular range *θ* = 5°–15°) gains energy much faster than the rest. This localised enhancement in the acceleration is driven by the jet of super-thermal electrons produced by the propagating portion of the laser pulse and collimated by a self-induced azimuthal magnetic field^26,27^. Figure 7e, f shows the transverse and longitudinal electrostatic fields, respectively, in the region of the jet, which is shown as a contour plot, at an example time of *t* = 0.1 ps. The very high electron current density within the jet produces a transverse electrostatic field, which attracts protons towards the laser axis, as shown schematically in Fig. 7e. Figure 7f shows that the magnitude of the dual-peaked longitudinal electrostatic field is maximum in the vicinity of the jet. As the interaction evolves, the sheath-accelerated protons are gradually pulled towards the jet and into the enhanced field region. Protons pulled into the RPA (or temporarily merged) field can gain enough energy to traverse the depression in the field structure and into the boosted sheath field region, allowing continued acceleration. As such, with increasing proton energy the centre of the beam changes from target normal to an angle closer to the laser axis, as shown in Fig. 7d. The energy dependence of this effect is shown in Fig. 2b (same simulation). Importantly, this figure shows that a similar energy-dependent steering is measured experimentally (albeit to a lesser extent than in the 2D simulation) and only for targets that undergo RIT.

**Fig. 8:**
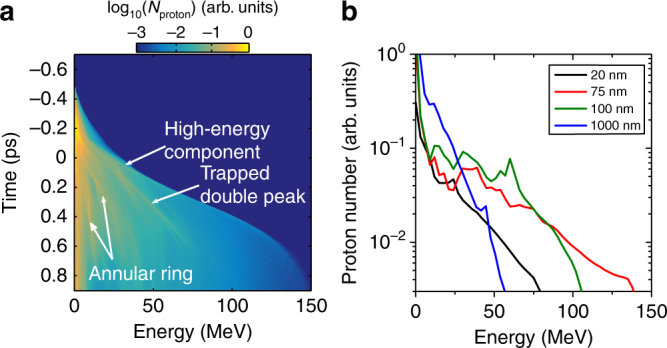
Energy spectra from the simulation results. **a** Temporal evolution of the proton energy spectrum for a $\ell$ = 75 nm target, sampled within an angular cone of ±60° of the laser axis. Distinct proton energy components are labelled. **b** Proton energy spectrum at *t* = 0.8 ps for given foil thicknesses $\ell$

Signatures of the various features described above are observed in the evolution of the proton energy spectra, as shown in Fig. 8a. Prior to RIT, the spectrum, sampled over a wide solid angle to enable comparison with experiment, is dominated by TNSA. As RPA begins just after *t* = −0.2 ps, a peak corresponding to the proton bunch trapped by the dual-field structure can be observed forming, part of which is further accelerated to energies higher than the sheath-accelerated protons. The remainder of the trapped bunch continues to gain energy before dissipating into a thermal spectrum late in the interaction (*t* ~ 0.6 ps). Similarly, spectral features resulting from the ring structure are also observed at low energy, but also degrade late in the interaction. The end result is an overall broad thermal spectrum with little evidence of distinct spectral features, similar to that measured experimentally, as shown in Fig. 2a. Figure 8b shows the proton spectra from the simulations at *t* = 0.8 ps for several target thicknesses. Similar to the experimental results in Fig. 2a, a thermal spectrum is observed, with a higher energy component emerging for thicknesses of the order of $\ell _{{\mathrm{opt}}}$. Note that the absolute energies in the simulations are higher than experiment because they are performed in 2D. The overall spectral shapes are very similar and are directly compared in Supplementary Fig. 1 and in the Supplementary Discussion.

### Scaling to multi-petawatt laser pulse parameters

**Fig. 9:**
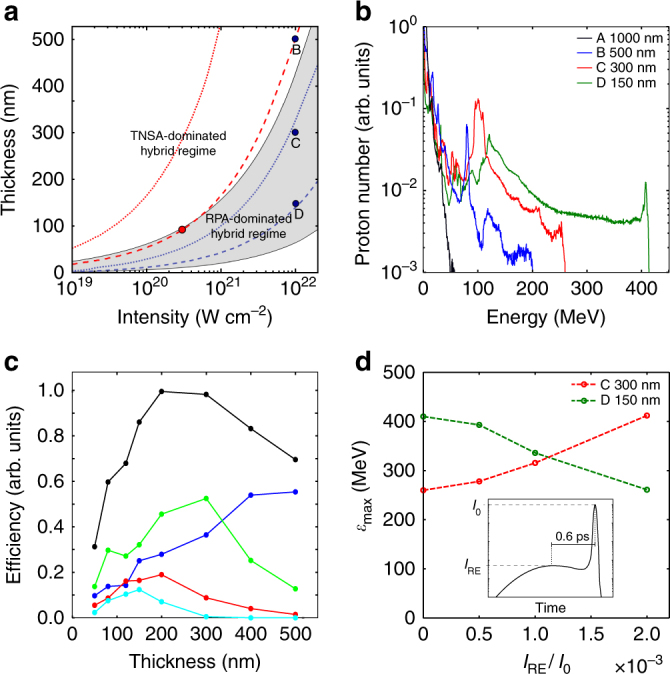
Modelling and simulation results for multi-petawatt scale laser parameters. **a** $\ell - I_{\mathrm{L}}$ parameter space for the TNSA- and RPA-dominant hybrid regimes, for plastic^18^. Scaling of the maximum $\ell$ for which RIT occurs (dotted) and optimum $\ell$ for which RIT occurs near the peak of the pulse (dashed) with *I*_L_, for pulse durations *τ*_L_ = 900 fs (red) and *τ*_L_ = 40 fs (blue). The red point corresponds to $\ell _{{\mathrm{opt}}}$ in the experiment. The blue points labelled B, C and D correspond to example cases discussed in the main text. **b** Proton energy spectra for four example target thicknesses, from 2D PIC simulations for 40 fs and 10^22^ W cm^−2^ laser pulses. **c** Laser-to-proton energy conversion efficiency as a function of $\ell$ (for the same laser pulse parameters). The total efficiency is shown (black), as well as the fraction in each of four energy bands: 10–50 MeV (blue); 50–150 MeV (green); 150–250 MeV (red); and >250 MeV (cyan). **d** Maximum proton energy as a function of the ratio of the intensity on the pulse rising edge, *I*_RE_, at an example time of 0.6 ps, to the peak laser intensity, *I*_0_. This is simulated by the addition of a wide, low intensity, pre-pulse, as illustrated in the inset, for the two example foil thickness cases C and D

In this section, we explore the broader relevance of RIT-enhanced hybrid acceleration and specifically the potential for this approach to be applied at multi-petawatt laser facilities under development. Figure 9a shows the $\ell$ parameter ranges over which the TNSA-dominant and RPA-dominant (shaded region) hybrid regimes occur as a function of laser intensity, *I*_L_, as calculated using the model in Qiao et al.^18^, for plastic density. In addition to the relatively long pulses explored experimentally in this work, we also consider short pulses typically achieved with Ti:sapphire lasers, and specifically choose 40 fs as an example pulse duration, which is routinely achieved with this type of laser and which is within the range expected to be delivered at a number of multi-petawatt laser facilities. The thickest target for which RIT occurs within the temporal window defined by ±*τ*_L_ (centred on the peak) is plotted as a function of intensity for *τ*_L_ = 900 fs and *τ*_L_ = 40 fs as dotted red and blue lines, respectively. This upper $\ell$ limit for the onset of RIT can be determined using the analytical model in ref. ^37^, although we find that the strict application of this model overestimates the degree of transparency when compared to experimental measurements (RIT occurs too early in the model). We compensated for this by using the maximum value of $\ell$ for which transmitted light is detected experimentally (for *I*_L_ = 3 × 10^20^ W cm^−2^) to adjust the onset time of RIT in the model and used that in the calculations of $\ell$ as a function of *I*_L_. A similar correction was applied for the *τ*_L_ = 40 fs case, based on the largest $\ell$ for which transmitted light is measured at that pulse duration, as reported in ref. ^38^. In both cases, the differences in target areal density are included in the calculations.

For the *τ*_L_ = 900 fs case, the optimum thickness for enhancing hybrid acceleration in the experiment ($\ell _{{\mathrm{opt}}}\sim$90 nm; the red point in Fig. 9a) corresponds to RIT occurring close to the peak of the pulse. The corresponding scaling of $\ell _{{\mathrm{opt}}}$ with intensity for *τ*_L_ = 900 fs and *τ*_L_ = 40 fs is plotted as the dashed red and blue lines, respectively. For the relatively long pulse case, this optimum condition coincides with the interface between the two hybrid regimes, at which the maximum ion velocities due to RPA and TNSA are approximately equal, over much of the intensity range. Thus, RIT-driven processes strongly influence the acceleration dynamics in the case of relatively long laser pulses, with the highest proton energies achieved via enhancement of the dual RPA-TNSA field structure. By contrast, for the *τ*_L_ = 40 fs case the thickness range for which RIT occurs at a given intensity is smaller, enabling RPA-dominant hybrid regimes with and without RIT enhancement to be accessible (below and above the dotted blue line in Fig. 9a, respectively). As discussed by Qiao et al.^18^, RPA-dominant hybrid acceleration may be advantageous in terms of producing ions beams with desirable RPA features, such as reduced energy spread.

To explore this further, we performed a series of simulations for laser pulses with *τ*_L_ = 40 fs and *I*_L_ = 10^22^ W cm^−2^, parameters achievable at next-generation, multi-petawatt laser facilities. These facilities are expected to deliver even shorter pulses and higher focused intensities when fully operational, but we choose conservative values, which could be achieved in the early stages of operation. By doing so we also avoid high field processes, such as radiation reaction, which are expected to influence ion acceleration (via the action of this force on the plasma electrons) at laser intensities of the order of 10^23^ W cm^− 2  ^^39–41^. Simulations were performed as a function of $\ell$ in the range 50–1000 nm.

Representative proton spectra are shown in Fig. 9b for four example thicknesses. Case A, $\ell$ = 1 μm, is in the TNSA-dominated thickness regime and the resultant spectrum is a thermal distribution with a relatively low maximum energy. As $\ell$ is decreased to 500 nm (case B), which is within the hybrid acceleration regime, but is too thick for transparency (Fig. 9a), the maximum proton energy increases and a narrow energy peak produced by RPA emerges (at ~80 MeV). For case C, corresponding to $\ell$ = 300 nm, a clear RPA-driven proton energy peak is observed, which together with the overall proton spectrum is boosted in energy by the onset of RIT. As $\ell$ is decreased down to 150 nm (case D), for which transparency occurs near the peak of the pulse, both the RPA and TNSA components are boosted to their highest observed energies, although the width of the RPA peak has increased significantly. For thinner targets, the proton energies decrease again, and below the lower bound $\ell$ of the hybrid regime low numbers of protons are accelerated by the rapid expulsion of the target electrons inducing Coulomb explosion of the foil. We note that as with the longer pulse and lower intensity case explored in the modelling discussed above, the 2D nature of the PIC simulations are likely to overestimate the achievable ion energies. Nevertheless, the overall trend of moving from a TNSA-dominated to a hybrid regime in which both RPA and TNSA occur, and finally to a regime in which this hybrid process is enhanced by relativistic transparency, is clearly observed as $\ell$ is decreased.

The corresponding total laser-to-proton energy conversion efficiency is plotted as a function of $\ell$ in Fig. 9c and shows an optimum thickness at 200 nm. The fractional conversion efficiency to four stated energy bands across the spectral range is also presented. There is a general decrease in the conversion to the lowest energy band and increase in the upper energy band as $\ell$ is decreased to 150 nm. Below this value, there is a decrease in the conversion to all parts of spectrum. The conversion efficiency to the second band, which contains the RPA peak, is maximised for $\ell$ ~ 300 nm. This is the thickness region at which the onset of relativistic transparency occurs late in the interaction (just below the upper bound $\ell$ limit for RIT in Fig. 9a), for these laser pulse and target density parameters. It is therefore in this thickness range that RPA is most effective (the RPA acceleration time is longest).

Finally, we also consider the influence of the laser intensity contrast on the acceleration dynamics. Suppression of the laser amplified spontaneous emission levels to achieve ultrahigh laser intensity contrast on the timescale of tens to hundreds of picoseconds prior to the peak intensity is a necessary first step to use foils of the thickness range considered here. However, this does not in itself enable control of the acceleration physics because, as discussed in Powell et al.^26^, the expansion dynamics of ultrathin foils are highly sensitive to the intensity of the picosecond rising edge of the laser pulse. This in effect changes the time during the interaction at which transparency occurs. Thus, $\ell _{{\mathrm{opt}}}$ depends on the intensity profile on the rising edge of the laser pulse. To illustrate this, the intensity level within a picosecond of the peak intensity was varied by the addition of a broad, low intensity pulse; the rising edge intensity at an example time of *t* = −0.6 ps is denoted *I*_RE_. The effect of varying *I*_RE_ on the maximum proton energy is considered for cases C and D in Fig. 9d. The maximum energy achieved in case D, which corresponds to $\ell _{{\mathrm{opt}}}$ for an ideal pulse, decreases because RIT occurs earlier in the interaction, thus decreasing the time over which RPA occurs. By contrast, the maximum proton energy increases for the $\ell$ = 300 nm case C target. This target is close to the threshold for RIT to occur (near the end of the interaction) for the ideal pulse (just below the dotted blue line in Fig. 9a) and therefore far from optimal for the transparency-enhanced hybrid acceleration scheme. With increasing *I*_RE_, the target expands faster, bringing the RIT time closer to the peak of the laser pulse interaction and thereby increasing the maximum proton energy. We note that the overall acceleration mechanism is the same, but the target thickness at which the maximum proton energy is achieved changes.

## Discussion

In summary, we report experimental and simulation results for proton acceleration in the interaction of ultraintense, linearly polarised laser pulses with ultrathin foils. Efficient acceleration of protons to energies exceeding 94 MeV is demonstrated. Simulation results show that the acceleration occurs via a dual-peaked electrostatic field, produced by a combination of the RPA and TNSA mechanisms, and that protons are efficiently accelerated in this hybrid RPA-TNSA scenario. RIT enhances the fields and produces a magnetically confined and directional jet of super-thermal electrons that drives higher proton energies over a relatively narrow angular range. The results demonstrate that by controlling the onset of RIT it is possible not only to enhance the maximum proton energy in the RPA-TNSA hybrid acceleration regime but also to manipulate the directional properties of the final proton beam.

Extrapolating the results via analytical and PIC modelling, RIT is expected to be important for relatively long pulses over a wide range of intensities achievable with present high-power lasers. In the case of short pulses and ultrahigh intensities planned for multi-petawatt laser facilities, several different regimes of hybrid acceleration occur. By tuning the target thickness, and potentially the pulse rising edge intensity profile, it is possible to transition between TNSA- and RPA-dominated hybrid schemes, and between regimes with and without RIT-driven enhancement (either side of the dotted blue line in Fig. 9a). As shown in Fig. 9b, with fixed laser pulse parameters, the resulting proton spectrum can thus be tailored to produce a RPA-driven spectral peak at mid-range energies (full width at half maximum (FWHM) energy spread equal to 6%, 18% and 34% for cases B, C and D, respectively), or to enhance the maximum and peak proton energies at the expense of the energy width of the peak.

These results illustrate the potential to use hybrid acceleration schemes to tune the spectral and spatial properties of the resulting proton beams, even in the absence of an ability to change the polarisation of the drive laser pulse, in the early stages of operation of next-generation, multi-petawatt laser facilities.

## Methods

### Experiment

The experiment was performed using the Vulcan laser at the Rutherford Appleton Laboratory. Pulses of *p*-polarised, 1.053 μm-wavelength light were focused using an *f*/3 off-axis parabolic mirror and reflected from a plasma mirror, using the arrangement shown in Fig. 1a. Reference ^26^ shows the measured intensity contrast and enhancement produced by the plasma mirror. The pulse duration was *τ*_L_ = (0.9 ± 0.1) ps (FWHM) and the energy after the plasma mirror was (210 ± 40) J, with 30% contained within the focal spot. The peak intensity was *I*_L_ = (3 ± 2) × 10^20^ W cm^−2^. The targets were thin planar plastic foils, with thickness $\ell$, in the range 10 nm–1.5 μm. Comparative Al targets were also used. The targets were irradiated at 30° with respect to the target normal, such that proton beam components directed along the laser and target normal axes could be spatially resolved at the detector, as discussed in ref. ^26^. The proton beam spatial-intensity distribution was measured in discrete energy slices by using stacked dosimetry (RCF) film, interleaved with Cu filters for activation measurements, positioned 5 cm downstream.

### Simulations

The simulations were performed using the fully relativistic, 2D EPOCH PIC code^33^. The targets were initialised as a uniform mixture of C^6+^ and H^+^ ions, both at 60*n*_crit_, neutralised by an appropriate density of electrons with initial temperature equal to 10 keV. The initial target thickness was varied over the range 20 nm to 1 μm. The boundaries of the simulation box were all defined as free-space. For the main set of simulations, reported in Figs. 2–8, the simulation box was 160 μm × 70 μm with mesh cell size equal to 5 nm × 12 nm. The incoming laser pulse temporal and spatial profiles were both Gaussian, with FWHM equal to 0.4 ps and 5 μm, respectively, and the peak intensity was equal to 2 × 10^20^ W cm^−2^. The shorter pulse duration, compared with the experiment, is justified because the ion energies tend to be exaggerated in 2D simulations, particularly in the RIT regime, due to the reduced degrees of freedom. This results in excessive ion expansion and impacts the interaction dynamics. By reducing the pulse duration, it is ensured that the peak of the laser field can interact with a more realistically expanded plasma. For the short pulse simulation runs in Fig. 9, the laser pulse temporal FWHM was decreased to 40 fs and the intensity was increased to 1 × 10^22^ W cm^−2^. The rising-edge intensity contrast simulations reported in Fig. 9d, were performed by adding a 0.4 ps (FWHM) pre-pulse with peak intensity varied in the range (0.5–2.0) × 10^19^ W cm^−2^, 0.6 ps before the main (40 fs; 1 × 10^22^ W cm^−2^) pulse.

### Data availability

Data associated with research published in this paper can be accessed at 10.15129/5f3448cf-6737-4e21-ae69-742ab8d8631c.

## Electronic supplementary material

Supplementary Information
