# Supplementary material for: Near-100 MeV protons via a laser-driven transparency-enhanced hybrid acceleration scheme
Source: Nat Commun. 2018 Feb 20;9:724. doi: 10.1038/s41467-018-03063-9 (PMC5820283; doi:10.1038/s41467-018-03063-9)
Supplement: Supplementary file 1 — Supplementary Information [file 41467_2018_3063_MOESM1_ESM.pdf]

Supplementary Figures

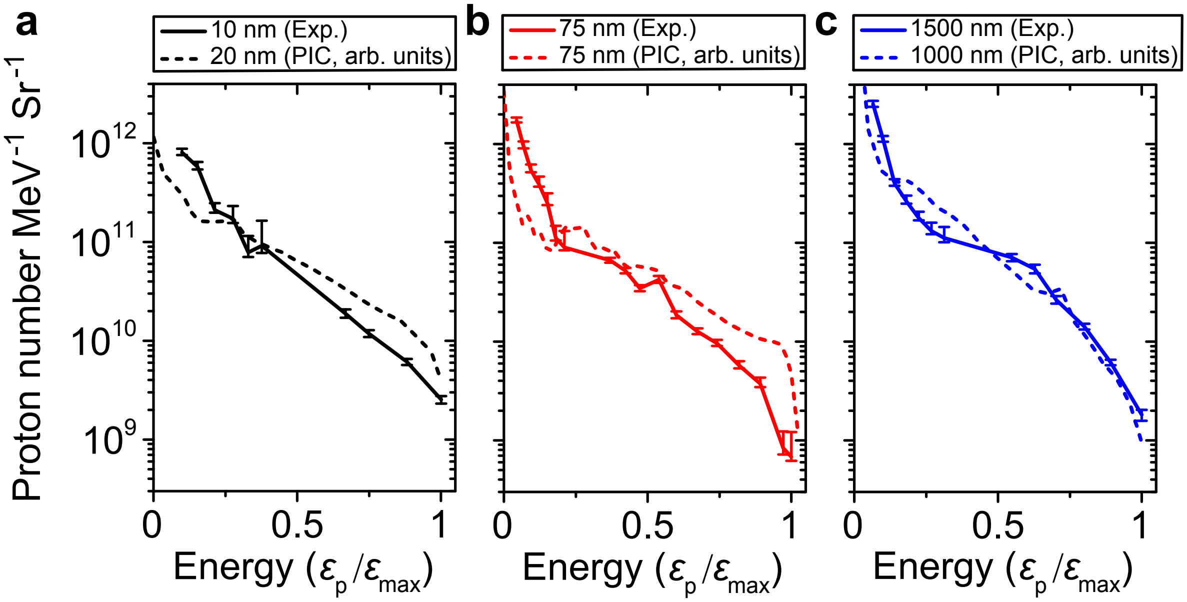

Supplementary Figure 1: Comparison of the proton energy spectral shapes. Measured proton energy spectrum and normalised spectrum from the PIC simulations described in the main paper, for: **a**, $\ell \ll \ell_{opt}$; **b**, $\ell \sim \ell_{opt}$; and, **c**, $\ell \gg \ell_{opt}$. The error bars are defined by the level of uncertainty in the calibration of the RCF.

Supplementary Discussion

In this Supplementary Information file, the shape of the proton energy spectra in the experiment and simulations are compared for targets of similar thickness. Supplementary Fig. 1 shows three example cases, corresponding to the optimum target thickness (ℓ_{opt}=75 nm) and targets which are much thinner and thicker. The spectra are plotted as a function of the maximum proton energy in each case and normalised to the proton flux. In all three cases, the overall shape of the measured spectra are in good agreement with the simulation results. The spectra are obtained at the end of the simulation (t=0.8 ps), when any significant peaks or other features have degraded. The temporal evolution of the simulation spectra are presented in the main paper, for the case ℓ=75 nm.
